# Involvement of CYP4F2 in the Metabolism of a Novel Monophosphate Ester Prodrug of Gemcitabine and Its Interaction Potential In Vitro

**DOI:** 10.3390/molecules23051195

**Published:** 2018-05-16

**Authors:** Yedong Wang, Yuan Li, Jia Lu, Huixin Qi, Isabel Cheng, Hongjian Zhang

**Affiliations:** College of Pharmaceutical Sciences, Soochow University, 199 Renai Road, Suzhou Industrial Park, Suzhou 215000, China; luckygoddesswyd@126.com (Y.W.); yuan722914@163.com (Y.L.); lujia0124@163.com (J.L.); 15105276692@163.com (H.Q.); isabel2018222@concordiashanghai.org (I.C.)

**Keywords:** gemcitabine, monophosphate prodrug, in vitro metabolism, CYP4F2, drug interactions

## Abstract

Compound-**3** is an oral monophosphate prodrug of gemcitabine. Previous data showed that Compound-**3** was more potent than gemcitabine and it was orally active in a tumor xenograft model. In the present study, the metabolism of Compound-**3** was investigated in several well-known in vitro matrices. While relatively stable in human and rat plasma, Compound-**3** demonstrated noticeable metabolism in liver and intestinal microsomes in the presence of NADPH and human hepatocytes. Compound-**3** could also be hydrolyzed by alkaline phosphatase, leading to gemcitabine formation. Metabolite identification using accurate mass- and information-based scan techniques revealed that Compound-**3** was subjected to sequential metabolism, forming alcohol, aldehyde and carboxylic acid metabolites, respectively. Results from reaction phenotyping studies indicated that cytochrome P450 4F2 (CYP4F2) was a key CYP isozyme involved in Compound-**3** metabolism. Interaction assays suggested that CYP4F2 activity could be inhibited by Compound-**3** or an antiparasitic prodrug pafuramidine. Because CYP4F2 is a key CYP isozyme involved in the metabolism of eicosanoids and therapeutic drugs, clinical relevance of drug-drug interactions mediated via CYP4F2 inhibition warrants further investigation.

## 1. Introduction

Over the last several decades, a number of nucleos(t)ide analogs have been successfully developed as antiviral, anticancer and antiplatelet drugs. Because unsubstituted nucleos(t)ides are often polar compounds with low membrane permeability, the prodrug strategy has been employed frequently to enhance pharmaceutical properties (such as permeability, metabolic stability and oral bioavailability), efficacy via organ-specific delivery, and safety via reduced formation of toxic metabolites [1]. One of the most successful prodrugs is sofosbuvir (GS-7977), a phosphoramidate prodrug of the β-d-2′-deoxy-2′-α-fluoro-2′-β-C-methyluridine nucleoside [2]. Another example among the antiviral agents is tenofovir alafenamide, a prodrug of (*R*)-9-(2-phosphonomethoxypropyl) adenine (tenofovir) [3]. Both sofosbuvir and tenofovir alafenamide belong to a class of prodrugs termed ProTides, a technology platform that is used to deliver high intracellular concentrations of respective nucleoside monophosphate and/or triphosphate [4,5,6].

For the anticancer drug gemcitabine (2′,2′-difluorodeoxycytidine), a number of drug delivery options, including prodrugs, have been explored to increase its oral bioavailability and improve efficacy/safety profiles [7]. With respect to gemcitabine prodrugs, the primary focus has been on the 4-amino group to reduce the first-pass effect due to deamination and the 5′-OH group, in order to enhance bioavailability and cellular delivery [8]. The protection of the amino group from the enzymatic deamination is exemplified by LY2334737, a valproic acid prodrug at the 4-N position [9]. At the 5′-postion, several prodrugs have been reported, including but not limited to fatty acid esters, cardiolipin conjugates, and phosphoramidate [7]. In particular, NUC-1031, a phosphoramidate prodrug (a ProTide) of gemcitabine, has been advanced to clinical trials with promising antitumor efficacy [10].

**Figure 1:**
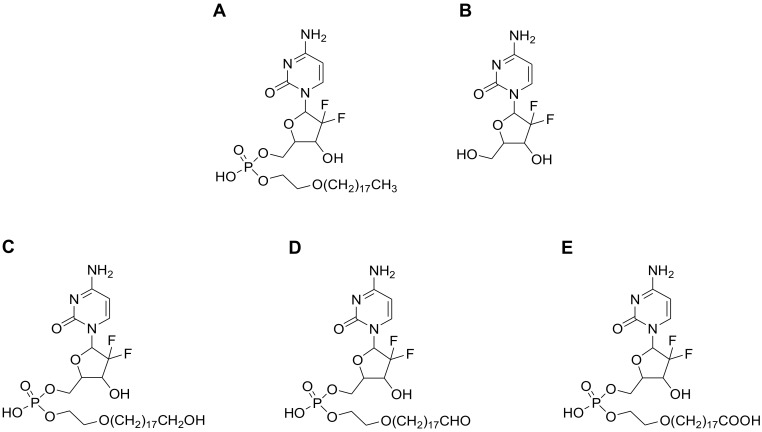
Structures of Compound-**3** and its proposed metabolites. (**A**) Compound-**3** (M0); (**B**) Gemcitabine (M1); (**C**) Hydroxyl metabolite (M3); (**D**) Aldehyde metabolite (M2); and (**E**) Carboxylic acid metabolite (M4).

In a previous report, we described a monophosphate ester prodrug of gemcitabine (Compound-**3**, Figure 1) with a unique hydrophobic tail at the 5′-position [11]. In vitro and in vivo testing in the preclinical setting revealed that the compound possessed an enhanced antitumor activity as compared to gemcitabine itself and LY2334737. In addition, Compound-**3** was orally active in the H460 tumor xenograft model (non-small cell lung cancer) and its cellular uptake had a low propensity to be limited by human equilibrative transporter 1 (hENT1), a nucleoside transporter that has been implied in gemcitabine transmembrane transport and certain tumor resistance [12].

To support its further development, we have been conducting a battery of preclinical studies to characterize drug-like properties of Compound-**3** with respect to absorption, distribution, metabolism and excretion. In the present study, we summarized in vitro metabolism of Compound-**3** using various matrices per regulatory recommendations. In particular, the involvement of cytochrome P450 4F2 (CYP4F2) was evaluated. CYP4F2 is an important CYP isozyme in the human liver, which is involved in the metabolism of endogenous substances as well as xenobiotics [13,14]. As such, CYP4F2 mediated interaction between Compound-**3** and pafuramidine was also investigated.

## 2. Results

### 2.1. Metabolic Stability of Compound-***3***

**Figure 2:**
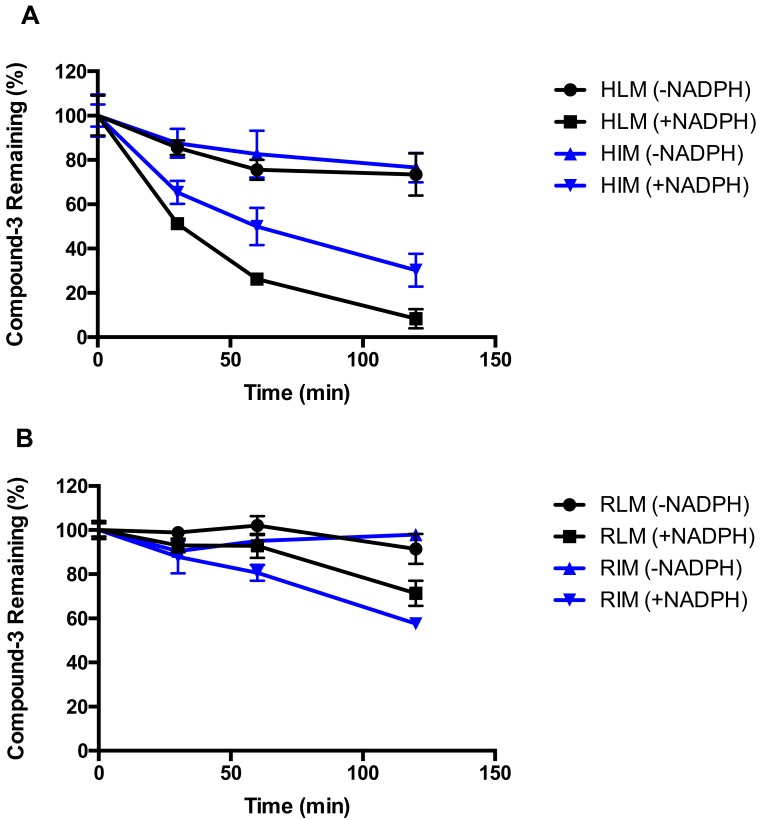

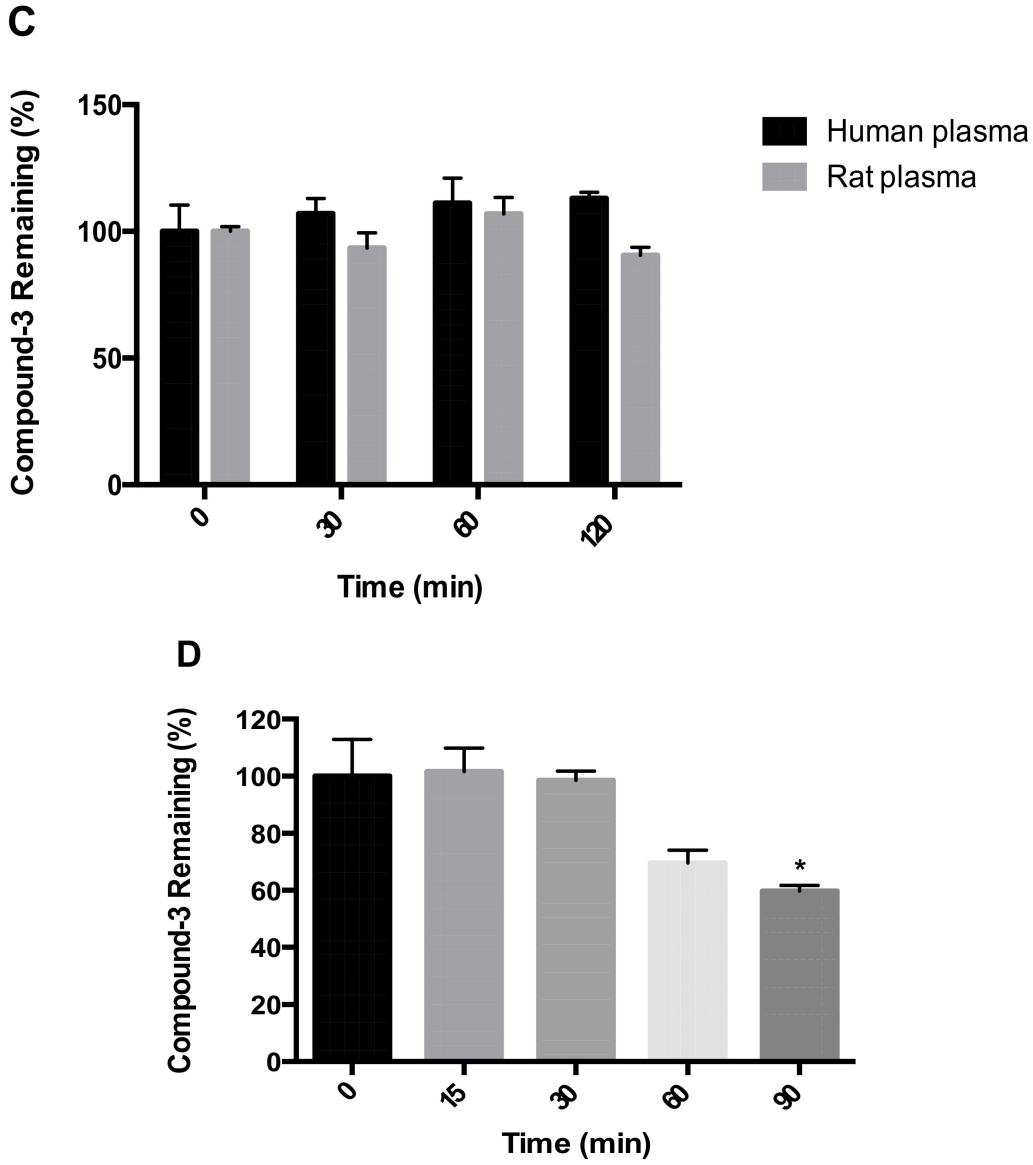
In vitro metabolic stability of Compound-**3**. HLM and HIM (**A**); RLM and RIM (**B**); Rat and human plasma (**C**); Human hepatocytes (**D**). Compound-**3** (1 μM) was incubated with liver microsomes (0.2 mg/mL) in the absence and presence of NADPH, plasma and hepatocytes (1 × 10^6^ cell/mL) at 37 °C (n = 3). * *P* < 0.05 vs. Compound-**3** remaining (100%) at 0 min.

Metabolic stability of Compound-**3** was examined in liver and intestinal microsomes from human and rat, plasma of human and rat, and human hepatocytes. As illustrated in Figure 2, Compound-**3** was relatively unstable in the presence of NADPH in both human liver and intestinal microsomes, with more than 70% and 50% disappearance after 60 min incubation (Figure 2A). In contrast, Compound-**3** showed moderate metabolism in rat liver (~10% disappearance in 60 min) and intestinal microsomes (~20% disappearance in 60 min) (Figure 2B). In the absence of NADPH, Compound-**3** was relatively stable. In addition, Compound-**3** did not display noticeable disappearance in human and rat plasma (Figure 2C). In human hepatocytes, metabolism of Compound-**3** was observed after 60 min incubation (~30% disappearance) (Figure 2D). The above results suggested that oxidative metabolism might be a major biotransformation pathway in those in vitro matrices recommended by regulatory agencies.

### 2.2. Detection of Compound-***3*** Metabolites

**Figure 3:**
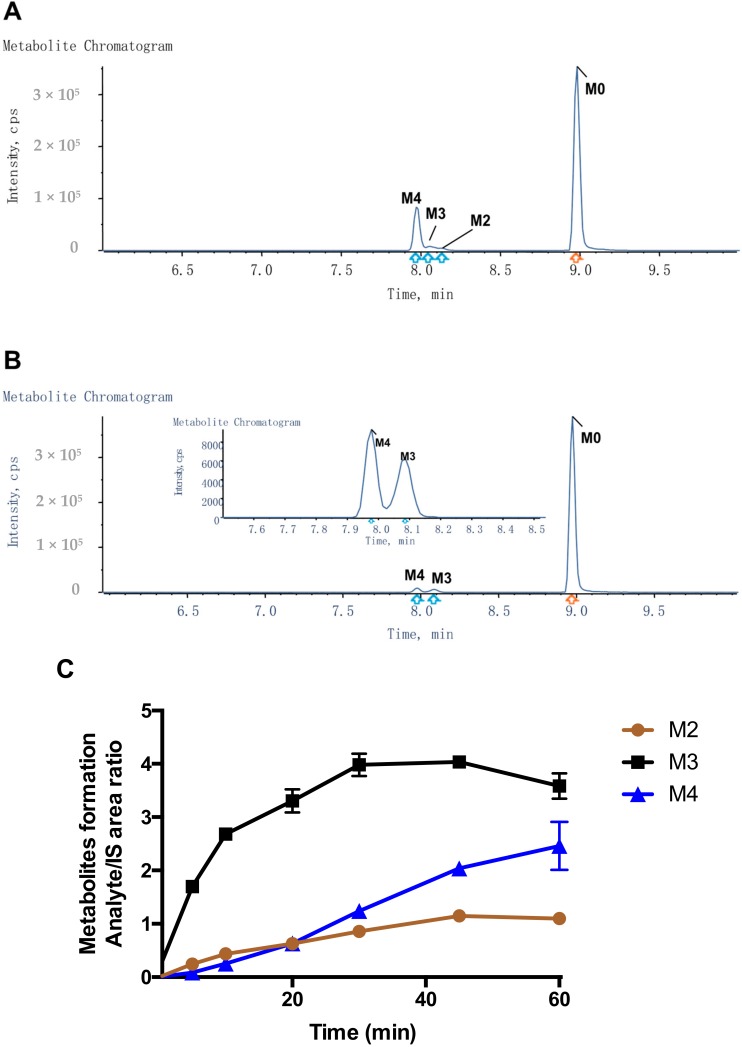

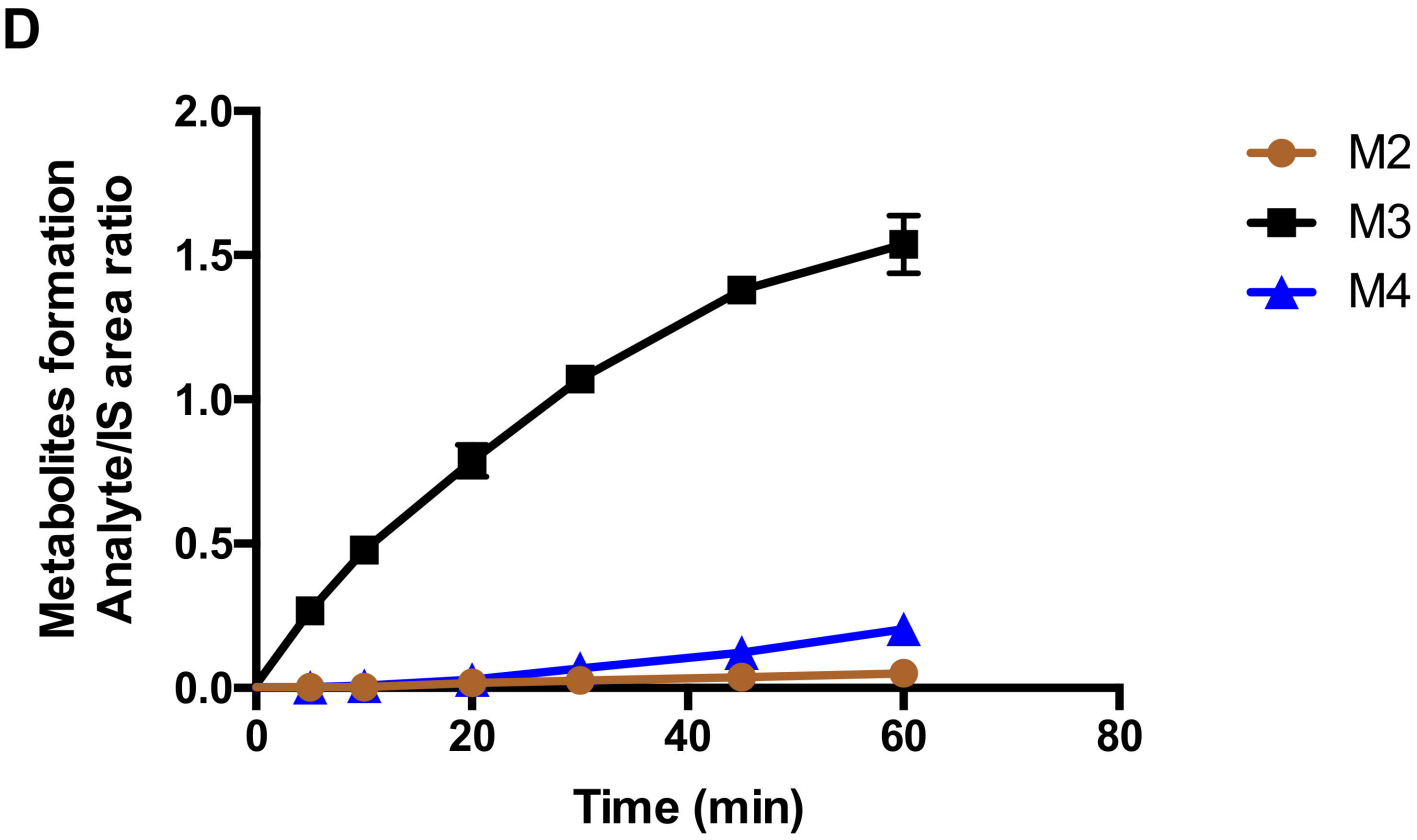
Detection of Compound-**3** metabolites in vitro. (**A**) Representative ion chromatogram in HLM; (**B**) Representative ion chromatogram in RLM; (**C**) Metabolite formation in HLM; (**D**), Metabolite formation in RLM. Metabolic profiles of HLM and RLM samples were collected after 60 min incubation of Compound-**3** (10 μM), liver microsomes (1 mg/mL) and NADPH at 37 °C (n = 3). For metabolite formation kinetics, Compound-**3** (1 μM) was incubated with HLM and RLM (0.2 mg/mL) in the presence of NADPH at 37 °C.

To detect metabolites of Compound-**3**, UPLC/Triple TOF 5600^+^ MS analysis was carried out for selected in vitro samples using the above matrices. Based on chromatographic retention and MS fragmentation behaviors of the parent compound, the HRMS technique was able to generate valuable structural information and characteristic fragmentation patterns. Figure 3 shows representative ion chromatograms from human and rat liver microsomal samples. Using accurate masses of respective ions, Compound-**3** (M0) could be detected with a retention time of 8.98 min and a protonated molecular ion [M + H]^+^ of *m*/*z* 640.353 in the positive scan mode. Similarly, three metabolites of Compound-**3** were identified: protonated ion [M + H]^+^ of *m*/*z* 654.333 (M2), *m*/*z* of 656.348 (M3), and *m*/*z* 670.328 (M4). Based on accurate masses of respective ions and fragmentation patterns, M2, M3 and M4 were designated as aldehyde, alcohol and carboxylic acid, respectively (Figure 1 and Table 1). Those metabolites could result from terminal oxidative metabolism of Compound-**3**.

Because of the lack of authentic metabolite standards, the absolute quantitation of M2, M3 and M4 could not be performed. Using Area/IS ratio, however, the formation of those metabolites could be traced. In human liver microsomes, incubation of Compound-**3** led to rapid increases of M3 (alcohol) at early time points (Figure 3C). M2 (aldehyde) also increased at early time points and then remained relatively unchanged. In contrast, the formation of M4 showed a linear increase over time, and a peak formation was not observed during the 60 min incubation. The above results suggested that Compound-**3** was oxidized to alcohol (M3), then dehydrogenated to aldehyde (M2), and finally oxidized to carboxylic acid (M4), which was the most stable form of all metabolites detected.

### 2.3. Hydrolysis of Compound-***3*** by Alkaline Phosphatase

**Figure 4:**
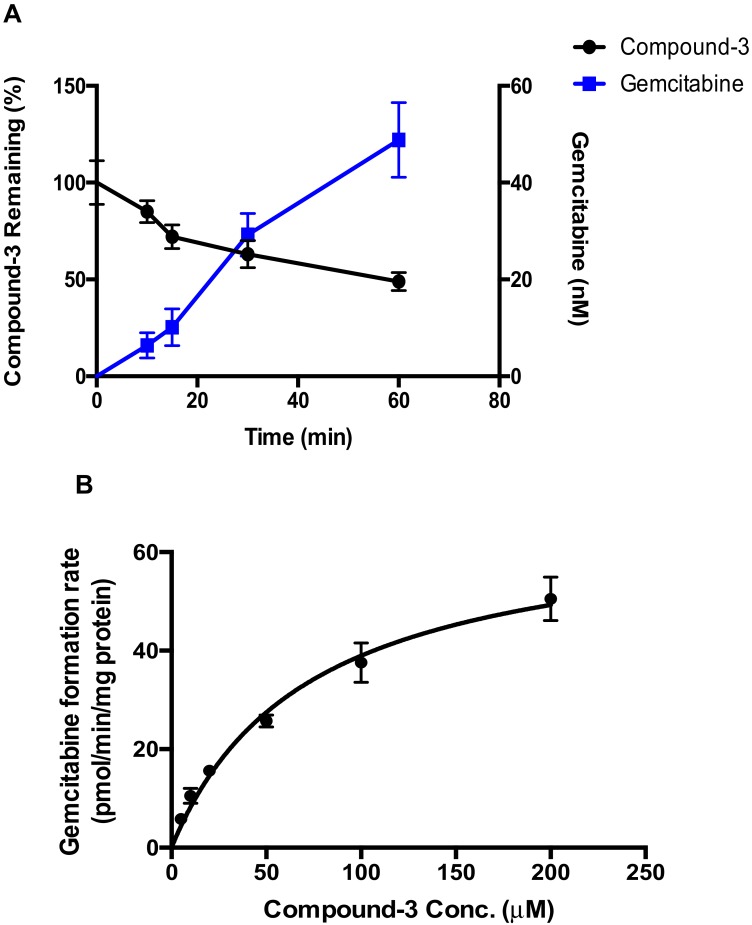
Hydrolysis of Compound-**3** by alkaline phosphatase. (**A**) Formation of gemcitabine. Compound-**3** (10 µM) was incubated with alkaline phosphatase (0.1 mg/mL) for different time points at 37 °C (n = 3); (**B**) Formation kinetics of gemcitabine by alkaline phosphatase (0.1 mg/mL) at various concentration levels for 60 min at 37 °C (n = 3).

Because Compound-**3** is a phosphate ester, hydrolysis of the ester bond might occur in various matrices tested. However, neither gemcitabine nor gemcitabine monophosphate were detected in Compound-**3** incubations using the HRMS technique as described above (Table 1). Alkaline phosphatase, a hydrolytic enzyme that shows high preference for phosphate esters [15], was therefore tested for the hydrolysis of Compound-**3**. As illustrated in Figure 4, the formation of gemcitabine resulting from the hydrolysis of Compound-**3** displayed a linear increase as a function of time (Figure 4A). Linearity was also observed with different alkaline phosphatase protein concentrations (Figure S1). Under optimal incubation conditions (0.1 mg/mL protein and 60 min incubation time), the formation of gemcitabine exhibited a typical Michaelis-Menten kinetics (Figure 4B), with K_m_ of 57.2 μM and V_max_ of 60.8 pmol/min/mg-protein, respectively.

### 2.4. Identification of CYP Isozymes Involved in Compound-***3*** Metabolism

**Figure 5:**
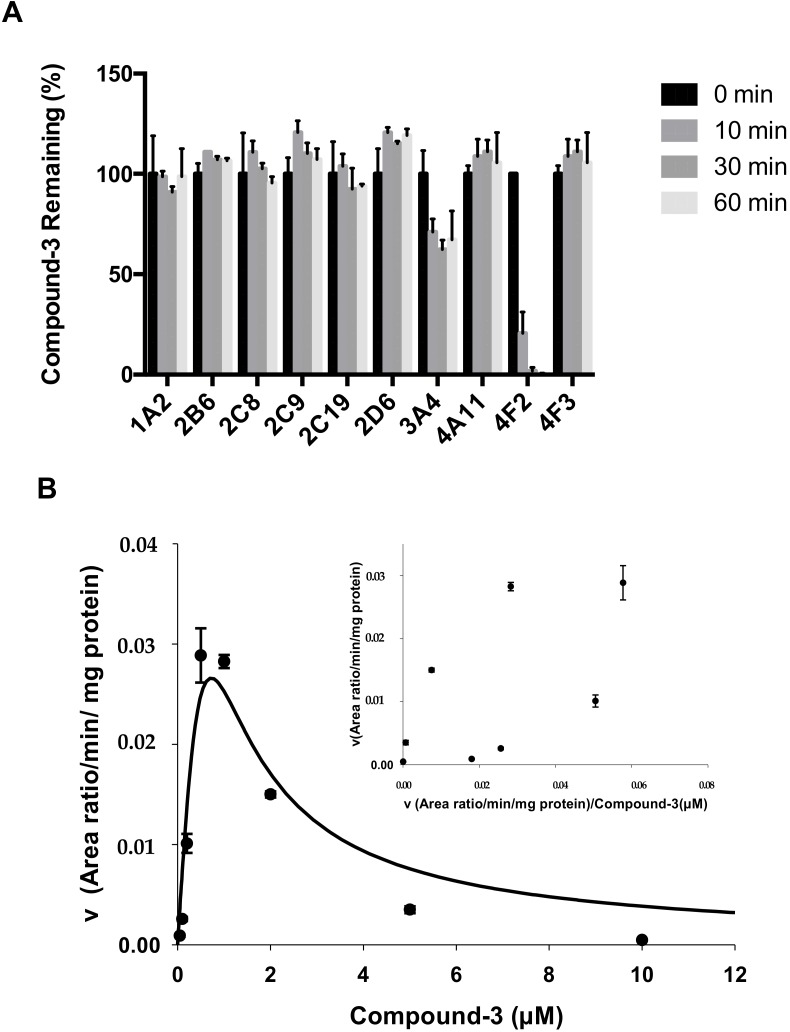
Metabolism of Compound-**3** by CYP isozymes. (**A**) Formation of M4 (carboxylic acid) in incubations of Compound-**3** (1 μM) with various CYP isozymes (50 nM) (n = 3); (**B**) Formation kinetics of M4 in CYP4F2 when Compound-**3** was incubated with CYP4F2 (10 nM) at various concentration levels for 10 min (n = 3); inset, Eadie-Hofstee plots.

CYP enzymes mediate the metabolic clearance of many drugs and xenobiotics. To determine which CYP isozyme was involved in the metabolism of Compound-**3**, major drug-metabolizing CYP isozymes along with CYP4A11, CYP4F2 and CYP4F3 were tested. As shown in Figure 5A, the disappearance of Compound-**3** was fastest in the presence of CYP4F2, with Compound-**3** barely detectable after 30 min incubation. In contrast, CYP3A4 showed moderate metabolic activity and the rest of the CYP isozymes did not participate in the metabolism of Compound-**3**.

Separate reactions with CYP4F2 revealed that M2 (aldehyde) and M3 (alcohol) peaked after 5 min incubation and then decreased rapidly, whereas M4 (carboxylic acid) peaked after 60 min incubation (Figure S2). Using M4 area/IS ratio, further incubations in the presence of CYP4F2 showed that the formation of M4 exhibited significant substrate inhibition (Figure 5B). Eadie-Hofstee plots for Compound-**3** revealed a unique kinetic profile, and the estimated kinetic parameters K_m_ and K_i_ values were 0.69 µM and 0.0012 µM, respectively.

### 2.5. CYP4F2 Mediated Interaction

**Figure 6:**
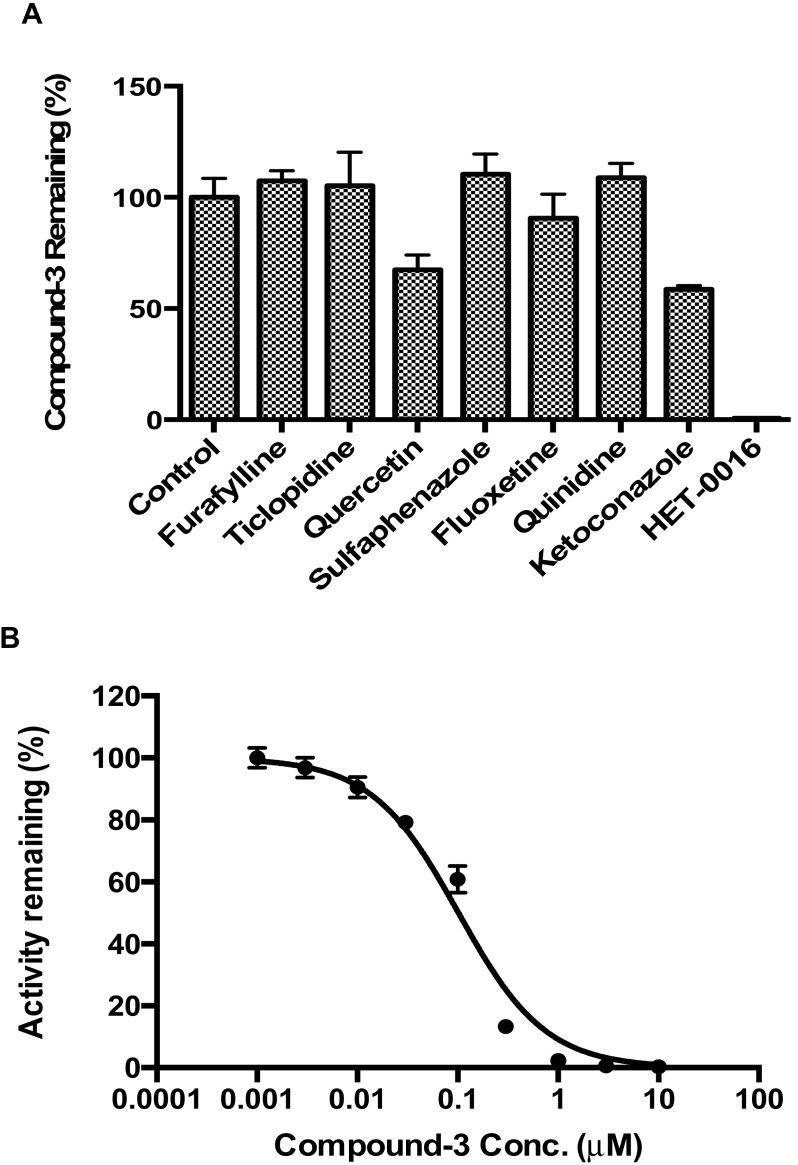

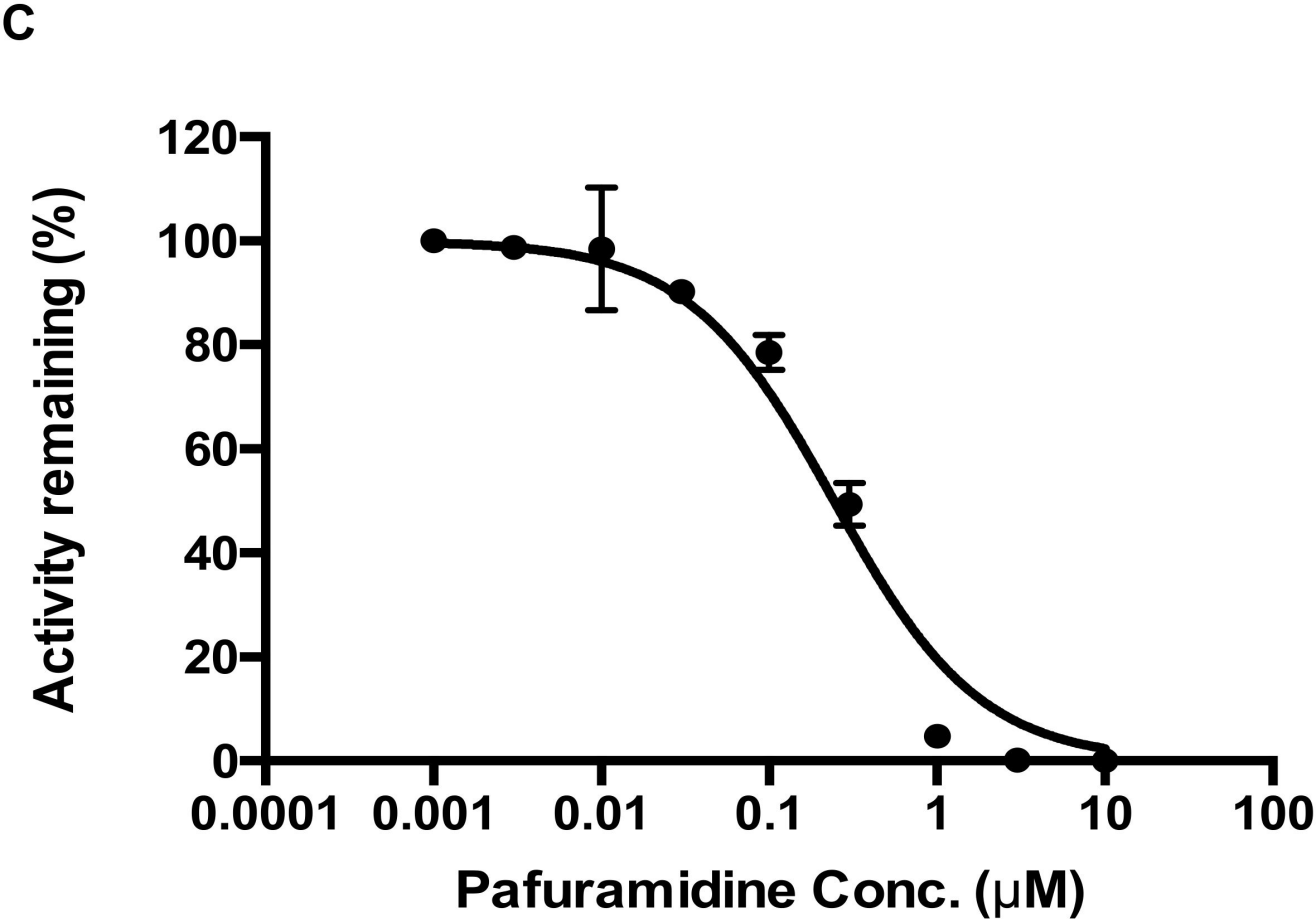
Inhibition of CYP4F2-mediated metabolism of Compound-**3**. (**A**) Inhibition of Compound-**3** metabolism in HLM by various chemical inhibitors; (**B**) Inhibitory effect of Compound-**3** on the metabolism of pafuramidine (1.5 μM) mediated by CYP4F2 (10 nM); (**C**) Inhibitory effect of pafuramidine on the metabolism of Compound-**3** (1 μM) mediated by CYP4F2 (10 nM). Data were average values of three separate incubations.

To further confirm the involvement of CYP4F2 in the metabolism of Compound-**3**, inhibition studies were carried out using specific chemical inhibitors of major CYP isozymes. As shown in Figure 6A, a complete inhibition of CYP4F2 was observed in the presence of HET-0016 (1 µM). In contrast, ketoconazole (a CYP3A4 inhibitor) and quercetin (a CYP2C8 inhibitor) displayed moderate inhibition and other inhibitors did not show noticeable effects. These observations were consistent with data described in Figure 5A.

As stated previously, CYP4F2 is involved in the metabolism of endogenous substances, as well as xenobiotics [13,14]. To test if Compound-**3** could affect the metabolism of therapeutic agents such as pafuramidine (a CYP4F2 substrate), CYP4F2-mediated drug interactions were investigated. As summarized in Figure 6B, Compound-**3** exhibited a strong inhibition of CYP4F2 mediated metabolism of pafuramidine with an IC_50_ value of 0.10 μM. When Compound-**3** was used as the substrate of CYP4F2 (Figure 6C), pafuramidine showed a strong inhibition toward Compound-**3** metabolism, with an IC_50_ value of 0.24 μM. These findings suggested that CYP4F2-mediated drug interactions might occur if pafuramidine and Compound-**3** were administered concomitantly. 

## 3. Discussion

Development of prodrugs follows similar research and regulatory paths to a typical small-molecule new chemical entity, except that a prodrug requires “activation” step(s) to release its active moiety. As described previously, Compound-**3** is a promising gemcitabine prodrug with oral antitumor activity [11]; subsequent studies have been carried out to support its development. Results from the present study indicated that alkaline phosphatase may be an important enzyme in the activation of Compound-**3**, a monophosphate prodrug of gemcitabine. With respect to metabolic clearance, in vitro metabolism studies using relevant matrices are an integral part of characterizing absorption, distribution, metabolism, and excretion (ADME), processes that are important in understanding the drug’s efficacy and safety profiles. The present study summarized findings from various in vitro matrices, and data suggested that CYP4F2 may play a critical role in the metabolic clearance of Compound-**3**.

Cytochrome P450 (CYP) enzymes belong to a superfamily of oxidoreductases that catalyze the metabolism of both endogenous substances and xenobiotics including drugs. With respect to drug metabolism, subfamily members of CYP1A2, 2C9, 2C19, 2D6, and 3A4 contribute to >70% of human CYP-mediated drug metabolism, with the largest fraction being catalyzed by CYP3A enzymes [16]. The CYP4F subfamily was first described as being involved in the hydroxylation of leukotriene B4, and hence playing an important role in modulating the concentrations of eicosanoids during inflammation [17]. CYP4F isozymes have also been shown to participate in the vitamin K ω-hydroxylation [18]. In the realm of drug metabolism, CYP4F isozymes, particularly CYP4F2, are involved in the metabolism of antiparasitic prodrug pafuramidine [19,20] and fingolimod (FTY720), a novel treatment for relapsing multiple sclerosis [21]. Because CYP4F isozymes amount to about 15% of the total hepatic CYP enzymes, and CYP4F2 displays polymorphic expression in certain human populations [13], the involvement of CYP4F2 in the metabolism of Compound-**3** observed in the present study could be clinically important. Metabolism of a lipid conjugate prodrug of tenofovir (CMX157, hexadecyloxypropyl tenofovir) by CYP4F2 has also been reported [22].

Another important aspect of preclinical metabolism research is the evaluation of the potential of CYP-mediated drug-drug interactions, because such information is useful in helping to design appropriate clinical studies and avoiding clinically relevant adverse events. Over the years, a number of reviews and guidelines have described in detail the conduct and values of non-clinical drug-drug interaction studies during development [23,24,25]. In the present study, we described for the first time that CYP4F2 could be involved in drug-drug interactions due to enzyme activity inhibition. In particular, both pafuramidine and Compound-**3** showed potent inhibition of CYP4F2-mediated metabolism (Figure 6). In addition, the formation kinetics of M4 (carboxylic acid metabolite) revealed a strong substrate inhibition profile (Figure 5B). The above findings suggest that further investigations are warranted to evaluate its clinical relevance, since the inhibition of CYP4F2 activity could lead to drug-drug or drug-endogenous substance interactions.

In summary, the present study describes key in vitro metabolic characteristics of Compound-**3**, a monophosphate prodrug of gemcitabine. Results suggest that alkaline phosphatase could be involved in its “activation”, whereas CYP4F2 is likely to play a role in its metabolic clearance. While Compound-**3** has exhibited promising oral antitumor activity and acceptable drug-like properties, the potential of CYP4F2-mediated drug-drug interactions remains to be confirmed in a clinical setting.

## 4. Materials and Methods

### 4.1. Chemicals and Reagents

Compound-**3** (purity ≥95% by HPLC) was supplied by PharmaResources, Ltd. (Shanghai, China). NADPH was purchased from Roche (Basel, Switzerland). Human liver microsomes (HLM, Cat. NO. 4133007, pooled from 20 different organ donors), pooled human intestinal microsomes (HIM), rat liver and intestinal microsomes (RLM and RIM) and recombinant human cytochrome P450s (CYP1A2, CYP2B6, CYP2C8, CYP2C9, CYP2C19, CYP2D6, CYP3A4, CYP4F2 and CYP4F3) were purchased from Corning Gentest (Woburn, MA, USA). Recombinant human P450 isoforms CYP4A11 was purchased from CYPex (Dundee, UK). Rat plasma was prepared in Laboratory Animal Center of Soochow University, and human plasma was supplied by Changzhou Fourth People’s Hospital. For rat plasma samples, male Sprague-Dawley rats were housed in a 12:12 light/dark cycle with standard chow and water ad libitum. Blood samples were collected in EDTA-containing tubes and plasma was obtained by centrifugation. Human blood samples were collected from non-cancerous subjects (no treatment history) with informed consent based on the Ethics Committee of Changzhou 4th People’s Hospital. Plasma was obtained via centrifugation and stored on ice for immediate use. Alkaline phosphatase was purchased from Sigma-Aldrich (St. Louis, MO, USA). Furafylline, quinidine, ketoconazole, ticlopidine, quercetin, sulfaphenazole, fluoxetine and HET-0016 were purchased from Absin Bioscience Inc. (Shanghai, China). Pafuramidine (purity ≥95%) was purchased from MedChemexpress (Monmouth Junction, NJ, USA). All other reagents and chemicals were of analytical grade and of the highest quality available commercially.

### 4.2. Metabolic Stability of Compound-***3*** in Various In Vitro Matrices

Stability of Compound-**3** was first investigated in microsomal systems from human and rat. The microsomal protein and Compound-**3** concentrations were optimized to 0.2 mg/mL and 1 µM, respectively. Before the start of the incubation, reaction mixtures containing phosphate buffer (100 mM, pH 7.4), microsomes and Compound-**3** in a total volume of 50 µL were warmed up at 37 °C for 10 min. The reaction was started with the addition of NADPH at a final concentration of 1 mM. Incubations were terminated at a specified time with the addition of 100 µL ice-cold acetonitrile containing the internal standard. The samples were then centrifuged, and aliquots of 10 µL of the supernatants were transferred into liquid chromatography-tandem mass spectrometry (LC-MS/MS) for the analysis of Compound-**3** levels.

For stability testing in plasma, the reaction was started with the addition of Compound-**3** (1 µM). At specified time points, aliquots of 25 µL of the reaction mixtures were transferred into 100 μL ice-cold acetonitrile containing the internal standard. The samples were then centrifuged, and aliquots of 10 µL of the supernatants were transferred onto LC-MS/MS for the analysis of Compound-**3** levels.

For stability evaluation in human hepatocytes, cryopreserved primary human hepatocytes were revived according to vendor’s protocol and diluted to a density of 1 × 10^6^ cell/mL with William’s Medium. The reaction was started with the addition of Compound-**3** (1 μM). At specified time points, aliquots of 25 μL of the reaction mixtures were transferred into 100 μL ice-cold acetonitrile containing the internal standard. The samples were then centrifuged, and aliquots of 10 μL of the supernatants were transferred onto LC-MS/MS for the analysis of Compound-**3** levels and its metabolites.

For metabolite identification, a higher concentration of Compound-**3** (10 μM) was incubated with liver microsomes (1 mg/mL) in the presence of NADPH (1 mM) and primary human hepatocytes at a density of 1 × 10^6^ cell/mL. Samples were collected at 0 min and 60 min with the addition of ice-cold acetonitrile. After vortexing and centrifugation, the supernatant was transferred into a new tube and evaporated to dryness. The samples were then reconstituted in 100 μL of water and methanol (*v*/*v*, 9:1). An aliquot of 10 μL of reconstituted solution was injected into UPLC/Triple TOF 5600^+^ MS for metabolite identification.

### 4.3. Hydrolysis of Compound-***3*** by Alkaline Phosphatase

Compound-**3** was incubated with alkaline phosphatase, and the linearity of gemcitabine formation as a function of time and protein concentration was first examined. The reaction mixtures consisted of alkaline phosphatase, 100 mM phosphate buffer (pH 7.4) and Compound-**3** (10 μM). The final volume of the incubation mixture was 200 μL. The mixture was warmed up at 37 °C for 2 min prior to the initiation of the reaction with the addition of the enzyme. At specified time points, 25 μL of the incubation mixtures was added to 100 μL cold acetonitrile containing the internal standard to terminate the reaction. Levels of gemcitabine were determined by LC-MS/MS. For enzyme kinetic evaluation, alkaline phosphatase protein concentration and incubation time were set to 10 μg/mL and 60 min, respectively, to ensure linearity of the formation of gemcitabine. Compound-**3** was tested at concentrations ranging from 0 to 200 μM.

### 4.4. Identification of CYP Isozymes Involved in Compound-***3*** Metabolism

Compound-**3** was incubated separately with human cDNA-expressed cytochrome P450s (CYP1A2, CYP2B6, CYP2C8, CYP2C9, CYP2C19, CYP2D6, CYP3A4, CYP4A11, CYP4F2 and CYP4F3). The incubation mixtures contained individual CYP isoenzymes (50 nM), potassium phosphate buffer (100 mM, pH 7.4), NADPH (1 mM), and Compound-**3** (1 µM). The final volume of the incubation mixture was 50 µL. Before the start of the incubation, reaction mixtures were warmed up at 37 °C for 10 min. The reaction was initiated with the addition of NADPH and the incubations were performed at 37 °C in a water bath. After 60 min of incubation, the reactions were terminated with an equal volume of ice-cold acetonitrile containing the internal standard. Control samples without NADPH but substrates were included. The samples were then centrifuged and aliquots of 10 μL of the supernatants were transferred onto LC-MS/MS for the analysis of Compound-**3** and M4 (carboxylic acid) levels.

In addition to Compound-**3** disappearance assays, effects of selective inhibitors of CYP isozymes were evaluated in human liver microsomes. The inhibitors included: CYP1A2 inhibitor furafylline (40 μM); CYP2B6 inhibitor ticlopidine (1 µM); CYP2C8 inhibitor quercetin (10 µM); CYP2C9 inhibitor sulfaphenazole (5 μM); CYP2C19 inhibitor fluoxetine (30 µM); CYP2D6 inhibitor quinidine (10 µM); CYP3A4 inhibitor ketoconazole (1 µM); and CYP4F2/3 inhibitor HET-0016 (1 μM). Incubation contained 0.2 mg/mL human liver microsomes, 100 mM phosphate buffer (pH 7.4), 1 mM NADPH, 1 μM Compound-**3**, and inhibitors at specified concentrations. Compound-**3** remaining was calculated based on concentrations after incubation in the presence or absence of respective inhibitors.

To investigate the formation kinetics of carboxylic acid metabolite (M4) from Compound-**3**, varying concentrations of Compound-**3** (0–10 µM) were incubated with 10 nM CYP4F2 and 1 mM NADPH in 100 mM phosphate buffer (pH 7.4). The reactions were carried at 37 °C for 10 min and terminated with the addition of ice-cold acetonitrile containing the internal standard. Samples were then centrifuged, and aliquots of 10 μL of the supernatants were analyzed by LC-MS/MS for M4 levels.

### 4.5. Evaluation of CYP4F2-Mediated Drug Interactions

CYP4F2-mediated drug interactions were examined in two separate setting: pafuramidine as the substrate and Compound-**3** as the inhibitor; and Compound-**3** as the substrate and pafuramidine as the inhibitor. The reaction system contained 10 nM CYP4F2, 100 mM phosphate buffer (pH 7.4), 1 mM NADPH, and substrate (pafuramidine 1.5 μM or Compound-**3** 1 μM). Inhibitor (Compound-**3** or pafuramidine) concentrations ranged from 0 to 10,000 nM. The incubation time was 5 min for pafuramidine and 10 min for Compound-**3**. Incubation temperature was 37 °C. LC-MS/MS analysis was performed as described above for the levels of DB775 or M4 (carboxylic acid metabolite of Compound-**3**). 

### 4.6. LC-MS/MS Analysis

Identification of Compound-**3** metabolites was carried out using Acquity UPLC/Triple TOF 5600^+^ system. Chromatographic separation for Compound-**3** and its metabolites were achieved by an Acquity UPLC HSS T3 column (100 × 2.1 mm i.d., 1.8 µM; Waters Corp.) maintained at 40 °C. The mobile phase was a mixture of 0.02% formic acid in 5 mM ammonium acetate (A) and methanol (B). The gradient elution was started from 2% B and maintained for 4 min, then increased linearly to 75% B over the next 4 min and maintained for 8 min. The gradient was rapidly increased to 95% B and maintained for 1 min and then reduced to 2% B and maintained at 2% B for 3 min to equilibrate the column. The flow rate was 0.4 mL/min and the eluent was monitored by UV detection at 280 nm.

For mass detection, a Triple TOF 5600^+^ mass spectrometer (AB Sciex, Framingham, MA, USA) was operated in positive ion electrospray (ES-positive) mode with the source temperature at 500 °C and scan range from *m*/*z* 100 to 1000 Da. Other parameters were set as follows: ion spray voltage, 5500 V; decluttering potential, 80 V; curtain gas, 40 psi; ion source gas 1, 55 psi; and ion source gas 2, 50 psi. Collision energies of 25 and 65 eV were used for TOF MS and product ion scans, respectively, and a collision energy spread of 20 was used in the MS/MS analysis. Information-dependent acquisition (IDA), together with real-time multiple mass defect filter, was used to trigger acquisition of MS/MS spectra.

Quantitative analysis of various analytes was achieved by a LC-MS/MS system consisting of an API4000 Qtrap mass spectrometer equipped with a turbo-V ionization source (Applied Biosystems, Foster City, CA, USA), two LC-20AD pumps with a CBM-20A controller, DGU-20A solvent degasser and a SIL-20A autosampler (Shimadzu, Columbia, MD, USA). HPLC column temperature was held at 40 °C. The flow rate was 0.3 mL/min and the total run time was 6 min. For Compound-**3** and DB775 analysis, an Agela Venusil XBP C18 column (50 × 2.1 mm; 5 μm particle size) (Bonna-Agela Technologies, Tianjin, China) was used to achieve chromatographic separation. The mobile phases used were 5 mM N-N-dimethylhexylamine, pH 6.8 (A) and acetonitrile (B) for Compound-**3**; 0.1% formic acid solution (A) and 0.1% formic acid in methanol (B) for DB775. For gemcitabine, an Agela Venusil MP C18 column (150 × 2.1 mm; 5 μm particle size) was used with the mobile phases of 5 mM ammonium acetate (A) and methanol (B). Conditions for M4 analysis were similar to that of Compound-**3**.

MS/MS quantitation was conducted using an API 4000 Qtrap mass spectrometer operated in the electrospray ionization positive mode with multiple reaction monitoring (MRM) to detect analytes and internal standards with a dwell time set to 100 milliseconds. The ion transitions monitored were Compound-**3**, 640.5 (M + H) → 246.0; Compound-**2** (internal standard for Compound-**3**) [11], 652.5 (M + H) → 344.1; M2, 656.3 (M + H) → 246.0; M3, 654.3 (M + H) → 246.0; M4, 670.3 (M + H) → 246.0; pafuramidine, 365.2 (M + H) →334.2; DB775 (metabolite of pafuramidine formed by CYP4F2) 351.1 (M + H) → 320.1; gemcitabine, 264.2 (M + H) → 112.0; diazepam (internal standard for pafuramidine and gemcitabine), 285.2 (M + H) → 193.2. Mass transition, collision energy and all other parameters were optimized for the best sensitivity. Data were collected and processed using the Analyst 1.5.2 data collection and integration software (AB Sciex, Framingham, MA, USA).

### 4.7. Data Analysis

Kinetic parameters of Compound-**3** in alkaline phosphatase were calculated using GraphPad Prism (Version 6.0, GraphPad Software Inc., San Diego, CA) and nonlinear regression analysis. Equation used was as the following (the Michaelis-Menten equation):

V = (V_max_ × [S])/(K_m_ + [S]) (1)

where V is the rate of metabolite formation, V_max_ is the maximum velocity, K_m_ is the Michaelis constant (substrate concentration at 0.5 V_max_), and [S] is the substrate concentration.

Kinetic parameters of Compound-**3** in recombinant human CYP4F2 were calculated using Enzyme Kinetics Modules of SigmaPlot (Version 14.0, Systat Software, San Jose, CA, USA). An uncompetitive substrate inhibition equation was also used:

V = (V_max_ × [S])/{K_m_ + [S] × (1 + [S]/K_i_)} (2)

where K_i_ is the disassociation constant describing the inhibitor-enzyme interaction.

## Figures and Tables

**Table 1:** Characterization of Compound-**3** metabolites in in vitro incubation systems by UPLC/Triple TOF 5600^+^ MS.

Name	Metabolic Reaction	Retention Time (min)	Observed Mass (*m*/*z*)	Calculated Mass (*m*/*z*)	Elemental Composition	Mass Error (ppm)	Matrix					
							ALP	HLM	RLM	HIM	RIM	Human Hepatocytes
M0	Compound-3	8.98	640.3538	640.3538	C29H52F2N3O8P	−8.8	√	√	√	√	√	√
M1	Gemcitabine	2.62	264.0782	264.0796	C9H11F2N3O4	0.2	√					
M2	Oxidation to aldehyde	8.14	654.3331	654.3331	C29H50F2N3O9P	0.8		√		√	√	√
M3	Oxidation to hydroxyl	8.05	656.3489	656.3487	C29H52F2N3O9P	−9.6		√	√	√	√	√
M4	Oxidation to carboxylic acid	7.97	670.3282	670.3280	C29H50F2N3O10P	−7.6		√	√	√	√	√

“√” metabolites were detected by high-resolution mass spectrometry. ALP, alkaline phosphatase; HLM, human liver microsomes; HIM, human intestinal microsomes; RIM, rat liver microsomes; RIM, rat intestinal microsomes.

## Supplementary Materials

The following are available online. Figure S1. Gemcitabine Formation from Compound-**3** Hydrolysis as a function of alkaline phosphatase protein concentrations. Compound-**3** (1 μM) was incubated with alkaline phosphatase concentrations ranging from 0.01–0.2 mg/mL at 37 °C (n = 3). Figure S2. Metabolites of Compound-**3** in human recombinant CYP4F2. Compound-**3** (1 μM) was incubated with human recombinant CYP4F2 (50 nM) in the presence of NADPH at 37 °C (n = 3).

## Abbreviations

CYP	cytochrome P450
HRMS	high resolution mass spectrometry
HLM or HIM	human liver or intestinal microsomes
IS	internal standard
RLM or RIM	rat liver or intestinal microsomes
