# Supplementary material for: Involvement of CYP4F2 in the Metabolism of a Novel Monophosphate Ester Prodrug of Gemcitabine and Its Interaction Potential In Vitro
Source: Molecules. 2018 May 16;23(5):1195. doi: 10.3390/molecules23051195 (PMC6100113; doi:10.3390/molecules23051195)
Supplement: Supplementary file 1 [file molecules-23-01195-s001.pdf]

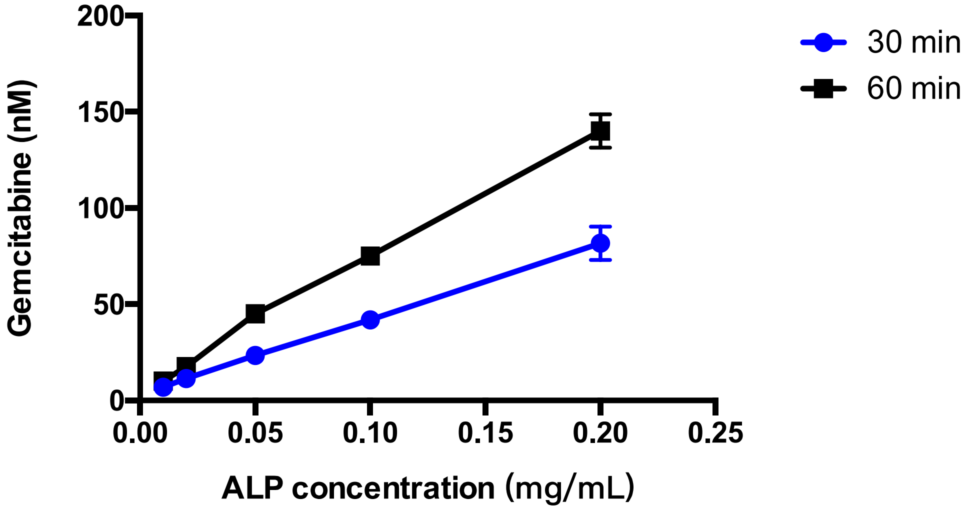

Figure S1. Alkaline phosphatase protein concentration-dependent formation of gemcitabine. Compound-3 (1 μM) was incubated with alkaline phosphatase concentration ranged from 0.01~0.2 mg/mL at 37 °C (n=3).

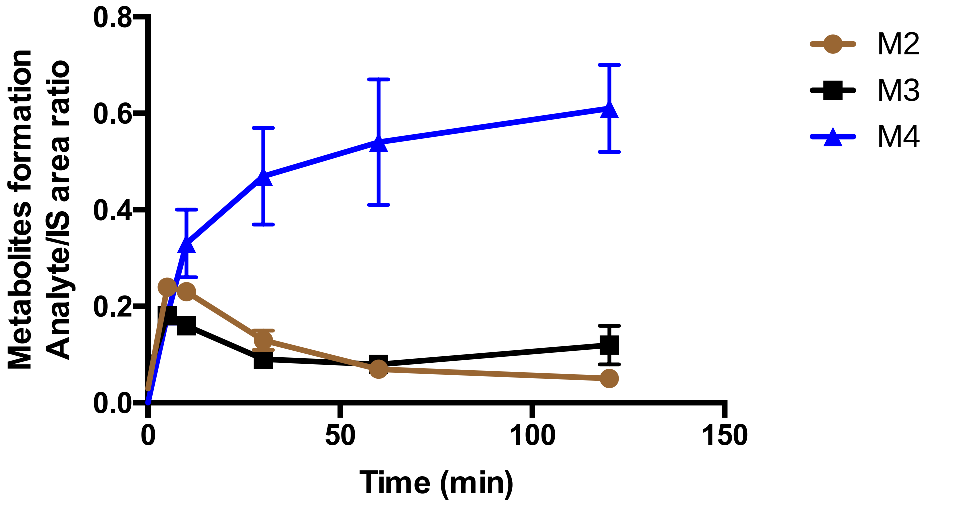

Figure S2. Formation of oxidative metabolites of Compound-3 in human recombinant CYP4F2. Compound-3 (1 μM) was incubated with human recombinant CYP4F2 (50 nM) in the presence of NADPH at 37 °C.
